# Mechanism of Selenium-Induced Inhibition of Arsenic-Enhanced UVR Carcinogenesis in Mice

**DOI:** 10.1289/ehp.10978

**Published:** 2008-02-01

**Authors:** Fredric J. Burns, Toby Rossman, Katherine Vega, Ahmed Uddin, Stefan Vogt, Barry Lai, Richard J. Reeder

**Affiliations:** 1 Department of Environmental Medicine, New York University School of Medicine, Tuxedo, New York, USA; 2 Experimental Facilities Division, Advanced Photon Source, Argonne National Laboratory, Argonne, Illinois, USA; 3 Department of Geosciences, Center for Environmental Molecular Science, State University of New York at Stony Brook, Stony Brook, New York, USA

**Keywords:** arsenic, cancer, mouse, prevention, radiation, selenium, skin, ultraviolet, UVR

## Abstract

**Background:**

Hairless mice that ingested arsenite in drinking water exhibited more than a 5-fold enhancement of ultraviolet radiation (UVR) carcinogenesis, whereas arsenite alone was carcinogenically inactive. Dietary organoselenium blocked the cancer enhancement effect of arsenic but not cancer induction by UVR.

**Objective:**

In this study we sought to explain selenium blockage of As enhancement by establishing the extent that As and Se tissue distributions are coincident or divergent.

**Methods:**

We used the X-ray fluorescence microprobe at the Advanced Photon Source (Argonne National Laboratory) to probe sections of skin and liver from hairless mice exposed to *a*) UVR, *b*) UVR + As, *c*) UVR + organoselenium, or *d*) UVR + As + organoselenium.

**Results:**

We found elevated levels of As in the skin epithelium (hair follicles and epidermis) and diffusely in the liver of mice exposed to UVR + As. Arsenic was entirely absent in skin in mice exposed to UVR + As + organoselenium, but a diffuse low level was seen in the liver. As and Se locations were consistently divergent in skin; As was more diffusely distributed, whereas Se was strongly associated with membranes. X-ray absorption near-edge spectra are consistent with the presence of the seleno-bis(*S*-glutathionyl) arsinium ion in the liver.

**Conclusions:**

Supplemental Se was uncommonly effective at preventing even a trace of As in skin at 14 or 196 days of continuous exposure to As in drinking water. Traces of the seleno-bis(*S*-glutathionyl) arsinium ion in the liver suggested that formation of this compound was more likely to be responsible for the As-blocking effect of Se than was a mechanism based on antioxidation.

In the United States, more than 1 million new cases of nonmelanoma skin cancer are diagnosed each year. Ultraviolet A (UVA) radiation and sunlight exposure are well-established risk factors leading to squamous cell and basal cell carcinomas (Urbach 2002). UV radiation (UVR) carcinogenesis is complex and not completely understood, but it seems likely to involve DNA damage, such as cyclobutyl pyrimidine dimers and 6,4-photoproducts combined with reactive oxygen species (ROS) associated with acute and/or chronic inflammation (Melnikova and Ananthaswamy 2005).

Paradoxically, arsenic is only weakly carcinogenic in the skin of laboratory animals (Goering et al. 1999). Recent evidence showed in mouse skin that arsenite [As(III)] is inactive as a carcinogen, but it powerfully enhances UVR carcinogenesis when added to the drinking water at concentrations < 5 mg/L (Burns et al. 2004; National Research Council 1999; Rossman et al. 2001, 2002). The possibility that the carcinogenicity of As in human skin could sometimes be the result of its enhancing activity cannot be ruled out based on current evidence (Yu et al. 2006).

Inflammation and its associated increase in ROS is frequently seen in skin exposed to UVR generally within 3–5 days after exposure (Hattori et al. 1996). ROS cause oxidative damage to DNA of cells exposed to either UVR or As (Hei et al. 1998; Kim et al. 2001; Nordenson and Beckman 1991; Shi et al. 2004). Increased ROS combined with a defective antioxidative defense system would leave cells particularly vulnerable to oxidative DNA damage (Karbownik et al. 2001; Ray and Husain 2002), although lifetime regimens of inorganic As in the drinking water of several mouse strains failed to induce neoplasias in any organ or strain (Gentry et al. 2004).

In mouse skin the antioxidants vitamin E and organoselenium blocked enhancement of UVR carcinogenesis by As (Rossman et al. 2001; Uddin et al. 2005), raising the possibility that oxidative DNA damage might be the underlying mechanism of the cancer-enhancing activity of As. The reduction of oxidative stress defenses by glutathione peroxidase knockouts, sensitivity to oxidant stress, and cancer in the lower gastrointestinal tract rise coordinately, suggesting a peroxidative stress contribution to carcinogenic susceptibility (Chu et al. 2004; Lewin et al. 2002; Schomburg et al. 2004).

The antioxidative activity of organoselenium compounds is believed to be based on the prominent role that selenium plays in many of the enzymes of the oxidative defense system (Burk et al. 2003; Gallegos et al. 1997; Hatfield and Gladyshev 2002; Lewin et al. 2002; Spyrou et al. 1996). However, whereas the cancer-enhancing effect of As was 100% blocked by dietary vitamin E or organoselenium [1,4-phenylenebis(methylene)selenocyanate (p-XSC)], only vitamin E inhibited the carcinogenicity of UVR; that is, organoselenium was ineffective against UVR (Uddin et al. 2005). This inconsistency raised the possibility that the action of organoselenium might have been based on a different mechanism than the well-established antioxidative effect of vitamin E. Unrelated studies in rabbits exposed to high levels of As and Se have established the existence of the seleno-bis(*S*-glutathionyl) arsinium ion {[(GS)_2_AsSe]^−^} in the liver and bile, which is a possible alternative way for Se to interfere with the activity of As (Gailer et al. 2002a, 2002b; Manley et al. 2006).

The purpose of the present study was to evaluate whether coincident or divergent tissue distributions at the cellular and subcellular levels in skin of mice fed As and Se might explain how Se blocks As cocarcinogenesis. We used the high spatial resolution and elemental sensitivity of the synchrotron X-ray fluorescence (XRF) microprobe at the Advanced Photon Source to track As and Se distributions in skin and liver.

## Materials and Methods

Hairless (HR-1) mice were obtained from Charles River Breeding Farms (Wilmington, MA). Beginning at time 0 (28 days of age), mice were exposed to 5 mg/L As(III) in drinking water, to 10 ppm p-XSC in rodent chow, or to both compounds and continued for 196 days. At 14 days, a topical fluence of 1.0 kJ/m^2^ UVR was started three times weekly and continued until 196 days. The p-XSC was mixed into powdered rodent chow, and controls were fed the unsupplemented powder. The UVR source was a bank of four Westinghouse FS-20 fluorescent tubes (Westinghouse Electric Corp., Pittsburgh, PA) mounted in parallel 15 cm apart at a distance of 30 cm from the skin surface. The fluence rate, as measured by an erythemal radiometer (model IL570; International Light. Inc., Newburyport, MA), was 0.2 kJ/m^2^ per minute. Each individual UVR dose was approximately one-fourth of the mouse minimal erythemic dose, that is, 1.0 kJ/m^2^. The mice were treated humanely with regard to suffering in conformity to rules established by the Institutional Animal Care and Use Committee of the New York University School of Medicine.

Tissue samples were prepared for elemental analysis at 14 days and 196 days. Samples of skin and liver were excised, fast frozen, and sectioned on a cryotome. Sections were mounted on silicon nitride support windows and desiccated before attachment to kinematic sample holders mounted on a motorized x–y-stage of a Leica DMXRE epifluorescence microscope (Leica Microsystems, Bannockburn, IL). Positional coordinates from selected locations were transferred to a corresponding x–y-stage for analysis of elemental distributions.

We used XRF microprobe analysis to examine element distributions and concentrations in tissue sections at beamline 2-ID-E of the Advanced Photon Source. We used an Si(111) single-bounce monochromator to tune the undulator synchrotron X-ray beam to 12.9 keV. The beam was focused into a 0.5-μm spot using Fresnel zone plate optics. For element distribution maps, the sample was rastered through the focal spot with a step of 1–2 μm and dwell time of 4 sec. A full energy-dispersive XRF spectrum was collected at each step using a Canberra three-element UltraLEGe detector (Canberra Instruments, Meriden, CT). Elemental maps were created by fitting the spectrum at each scan position with modified Gaussians using MAPS software (Vogt 2003). Area concentrations for elements were calculated by comparing integrated intensities of fluorescence peaks with those from thin film standards (NBS 1832 and 1833; National Bureau of Standards, Gaithersburg, MD).

We obtained X-ray absorption near-edge spectra (XANES) at beamline 2-ID-D in fluorescence mode using a Si-drift detector. Spectra were obtained at As and Se K-edges for the liver sections only of the As + Se exposure because no As was found in other tissues. Multiple spectra were obtained (up to 17) and then averaged. Spectra were also acquired for sodium arsenite, disodium arsenate heptahydrate, sodium selenite, and sodium selenate as oxidation state references.

## Results

**Figure 1:**
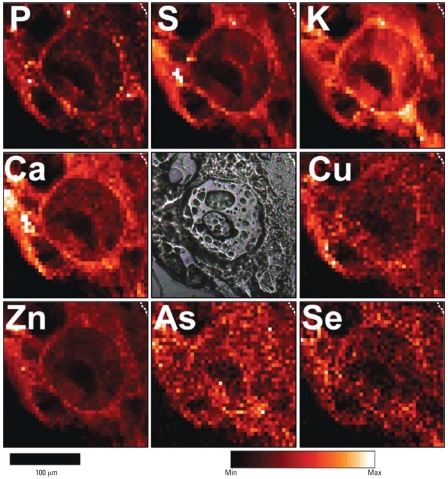
Micro-XRF element maps of hyperplastic epidermis taken from the skin of a mouse exposed to UVR and As(III) for 182 days. Dashed lines in upper right corners demarcate the boundary between epidermis and a tiny amount of dermis. Abbreviations: Max, maximum; Min, minimum. A phase-contrast image in the central panel shows a large, circular nucleus containing a large nucleolus. Other pocklike structures are artifacts of desiccation. The giant cell is a typical “sunburn” cell with a huge nucleus and a barely perceptible cytoplasm. The nucleolus, being mostly RNA, contains little, if any, of the sampled elements. Near the large cell are several normal-sized epidermal cells. The elements sulfur, phosphorus, potassium, calcium, copper, zinc, and Se are generally concentrated in the membrane regions at the peripheries of the cells.

The Advanced Photon Source provided opportunities to scan tissue sections to establish the distribution of metals and other elements and to make relatively long counts at specific loci for constructing comparative concentrations of the various elements. Figure 1 shows a region of hyperplastic mouse epidermis after 182 days of UVR treatment and 196 days of 5.0 μg/L of As only in drinking water. The large circular ring at the center of the field is the periphery of a giant nucleus, as typically found in sunburned regions of skin. Giant nuclei are a result of multiple DNA replications without cell division. Eventually these cells contain almost all DNA and very little cytoplasm, as suggested in these scans, where a clear demarcation between cellular and nuclear membrane is lost. The dark region within the giant nucleus is a nucleolus (seen in the microscope image in the central panel), which contains few, if any, of the sampled elements, as expected from a structure consisting primarily of RNA. Normal-sized keratinocytes seen below and to the left of the giant cell also contain dark nucleoli.

**Figure 2:**
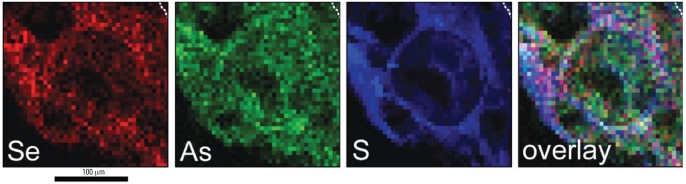
Contrasting false-color micro-XRF element maps corresponding to the S, As, and Se panels from Figure 1. Dashed lines in upper right corners demarcate the boundary between the epidermis and a tiny region of dermis. These images emphasize the differing subcellular distributions of As and Se in mice given As(III) only, particularly in the overlay, where As is clearly evident throughout the nucleus (excluding the nucleolus), whereas Se is concentrated more in the peripheral regions of the cells, where cellular and nuclear membranes tend to be located in these heavily UVR-damaged cells.

Several markers of membrane localization shown in Figure 1 are zinc, phosphorus, sulfur, potassium, and calcium. Similarly, Se shows a close association with membrane localization, which is consistent with the Se being associated with antioxidative enzymes that reside primarily in cellular membranes. Arsenic, by contrast, is more uniformly distributed than is Se, although overlap does occur in the membrane regions. Figure 2 shows contrasting color scans of the same field as in Figure 1 for As, Se, and S plus an overlay for better visualization of comparative localizations. The green color of As is evident throughout each nucleus in a generally uniform pattern, and little evidence of Se can be found in central regions. The disparity of the distributions suggests that As and Se are coexisting in the same cell with little or no tendency to form a complex; that is, Se associated with membrane enzymes is apparently not available to complex with As.

**Figure 3:**
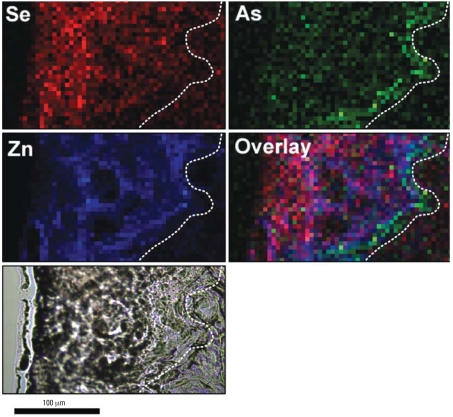
Micro-XRF element maps showing the distribution of As, Se, and Zn within the skin of a mouse exposed to UVR + As(III) for 182 days. Dashed lines demarcate the boundary between the epidermis (left) and the dermis (right). The highest concentrations of As are seen as a diffuse green line on the epidermal side of the demarcation corresponding roughly to the location of the proliferating epidermal basal cells. In contrast, the Se concentration is low near the basal layer and rises in epidermal regions closer to the skin surface. Colors are arbitrarily assigned to the elements, and intensity is scaled to maximum observed concentrations: Se = 0.007 μg/cm^2^, As = 0.004 μg/cm^2^, and Zn = 0.195 μg/cm^2^.

Another view of the disassociation between the distributions of As and Se is shown in Figure 3 at a lower magnification of an epidermal region from a mouse exposed to As only. The epidermis is highly hyperplastic and extends from the basement membrane (dashed line) to the surface keratin, a distance of about 125 μm or roughly 15 cell diameters. To the right of the dashed line is dermal tissue that is generally devoid of Zn and other elements because of low cellular content. The As is clearly associated most strongly with the epidermal basal layer that runs in a jagged route from the lower middle of the As panel to the upper right corner. Also in Figure 3, Se, by contrast, is low in the basal layer region but increases in the direction of the skin surface (i.e., at the far left).

**Figure 4:**
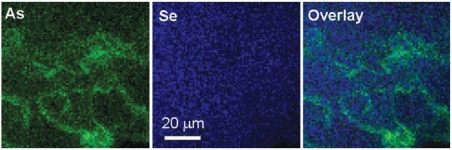
Micro-XRF element maps for As and Se and an overlay of As + Se for a dermal region of a mouse exposed to As(III) for 14 days and showing uptake of As into hair follicles. Colors are arbitrarily assigned and intensity is scaled to maximum observed area concentrations of As = 0.011 μg/cm^2^ and Se = 0.010 μg/cm^2^. The swirls and ovoid patterns are typical of follicle fields cut in cross section. These images show that As concentrated in the follicular epithelium is approximately the same as in the epidermal basal layer and that Se is relatively uniformly distributed throughout the dermis.

Figure 4 shows micro-XRF element maps for As and Se for a region of the dermis well below the epidermis containing cross sections of hair follicles from a mouse exposed to As only for 14 days. The complex pattern of As concentration is typical of hair follicles seen in cross section and establishes that the follicles take up As about equally to the basal cells (not shown). The Se in this region is a relatively low but uniform concentration, a distinct departure from the pattern for As.

**Figure 5:**
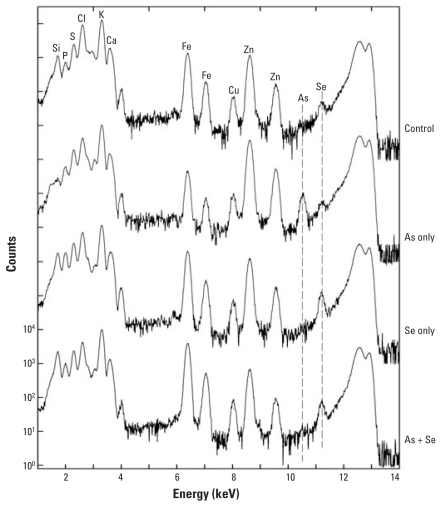
Micro-XRF spectra of skin from mice exposed for 14 days to As(III) only, Se(IV) only, or As(III) + Se(IV) compared with control. Abbreviations: Cl, chlorine; Fe iron. The control shows no As and a small Se peak. A definitive As peak is detected in the As-only skin just to the left of the Se peak. The Se peak is increased by a factor of about 2 in the Se-only and the As + Se skin. In the As + Se skin, the As peak is completely missing. Clearly, the As has been blocked from entering the skin by the Se even at this early time point.

**Figure 6:**
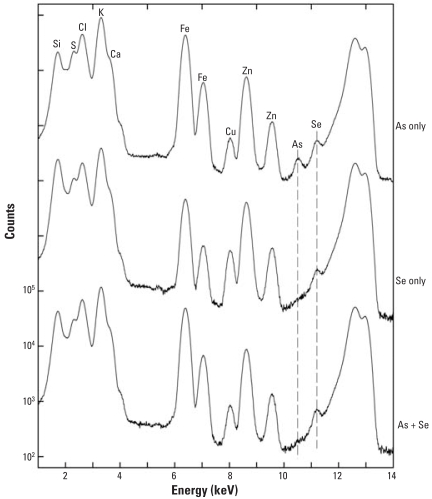
Micro-XRF spectra of skin exposed for 182 days simultaneously to UVR + As(III) only, UVR + Se(IV) only, or UVR + As(III) + Se(IV). Abbreviations: Cl, chlorine; Fe iron. The integrated spectra shown are averaged from the area scans ranging from 100 to 200 μm in size. Again, the As peak is apparent in the As-only skin and is somewhat lower relative to Zn than at the 14-day point, indicating little, if any, buildup of As between 14 and 196 days. Still, the As peak is completely eliminated by the presence of the Se even in the long-term exposure, as shown in the lowermost spectrum.

Figures 5 and 6 show micro-XRF spectra of skin from mice exposed for either 14 days or 196 days, respectively, to As only, Se only, As + Se, or neither As nor Se (control). The mice supplemented for 182 days were also exposed three times weekly to UVR beginning 14 days after As supplementation started. Se supplementation for 14 days may have increased the skin Se slightly, but heterogeneity among spots made it difficult to draw quantitative conclusions. Figure 6 also shows that Se supplementation for 196 days failed to increase Se levels in skin. This is not surprising, because mouse skin contains comparatively high background levels of Se.

**Figure 7:**
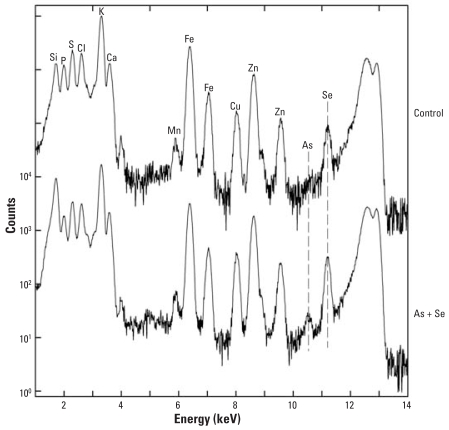
Micro-XRF spectra of liver from a control mouse and a mouse exposed for 14 days to As(III) + Se(IV). Abbreviations: Cl, chlorine; Fe, iron. No UVR was applied to these mice, because in the longer-term experiments, UVR was started 14 days after the start of the As to allow for buildup. Here the endogenous Se level is quite a bit higher relative to Zn compared with the level seen at 196 days and is increased only marginally by supplemental Se. However, even in the presence of Se, a small As peak was detected.

The spectra in Figure 6 demonstrate that no detectible As was present in mice given Se only and that a clear As peak was apparent in the mice given As only. The similarity between skin As concentrations at 14 versus 196 days indicates that the buildup of As was fairly rapid and that very little, if any, accumulation of As occurred during the longer exposure. Also clear is that the As peak is missing in mice given the Se supplement, which indicates that Se completely blocked uptake of As by the skin. The micro-XRF spectra in Figure 7 from liver of mice exposed to As + Se for 14 days show the presence of both As and Se and indicates that despite the Se supplement, some As was present in the liver.

**Figure 8:**
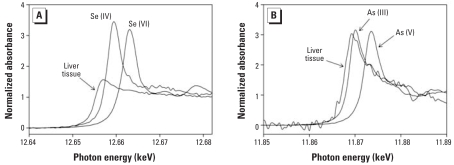
Se (*A*) and As (*B*) K-edge XANES from liver of a mouse fed As + Se for 14 days. Corresponding spectra for Se(IV) and Se(VI) (*A*) and As(III) and As(V) (*B*) oxidation state standards are also shown. The energy shifts relative to oxidation state standards seen in the mouse liver are similar to observations made in rabbit liver by Gailer et al. (2002b) at much higher As concentrations and suggest that some As is likely present as [(GS)_2_AsSe]^−^.

Normalized Se and As K-edge XANES from liver of a mouse fed As + Se for 14 days are shown in Figure 8. The low concentrations of these elements, especially for As, resulted in spectra with significant noise, even following aggregation of multiple scans. We also compared the normalized Se K-edge (Figure 8A) and As K-edge (Figure 8B) XANES with oxidation state standards. The Se absorption edge occurs at lower energy than for either the selenite [Se(IV)] or selenate [Se(VI)] standards. This shift in edge position relative to Se(IV) is similar to that observed for reduced Se bound with glutathionyl, specifically the compounds [(GS)_2_AsSe]^−^ and selenoglutathione [Se(GS)_2_] (Gailer et al. 2000) and in bile from rabbits exposed to As(III) (Gailer et al. 2002b). The distinctive peak in the edge region of the Se(IV) and Se(VI) standards is notably absent in the spectrum from the mouse liver, which was also noted for glutathionyl-bound reduced Se (Gailer et al. 2000).

The As absorption edge from mouse liver (Figure 8B) is displaced to lower energy by about 1.5 eV relative to the As(III) standard, using the inflection point as a reference, and significantly more relative to the arsenate [As(V)] standard. The difference relative to the As(III) standard is similar to the shifts observed for As(III) in the glutathionyl compounds [(GS)_2_AsSe]^−^ and arsiniumglutathione [As(GS)_3_] and suggests the presence of the same oxidation state for As in mouse liver as was seen in rabbit liver and bile (Gailer et al. 2002b; Manley et al. 2006).

The low signal/noise of both the Se and As spectra (resulting from their low concentrations) limits more detailed comparisons with published results. The As and Se concentrations used in the present study were chosen to correspond to levels at which carcinogenic enhancement has been observed in mice. Consequently, even though the observed spectra shown here are broadly consistent with those observed at much higher levels in rabbits, the results are not quantitatively sufficient for a definitive identification to be made. Nevertheless, the XANES results are consistent with the presence of [(GS)_2_AsSe]^−^ in the liver, as would be expected if supplemental Se has acted to block As from peripheral locations, such as skin.

## Discussion

The data presented here support the idea that a dietary supplement containing sufficient Se can block the cancer-enhancing activity of As in drinking water by preventing the As from getting to peripheral tissues. A likely way this could happen is by the occurrence of a reaction between As and Se in the presence of glutathione, probably in the liver, forming the reaction product [(GS)_2_AsSe]^−^, which is then eliminated from the body in the bile (Manley et al. 2006). Blocking As in this way is expected to be far more effective at preventing the carcinogenic or other toxic actions of As than, say, an oxidative stress reduction, because the As is effectively cleared out of the body. The practical import of these findings is that almost complete protection against As can be accomplished by an Se supplement at roughly double the concentration in food as the As concentration in the drinking water. Additionally, to the extent that Se participates in formation of the As–Se reaction product, this approach tends to mitigate any possible toxicity of the Se supplement, as well.

The blockage afforded against As by Se was equally effective at 14 days of exposure as at 196 days, implying that the effectiveness of the inhibition occurs early and persists for long periods of time in the absence of noticeable toxicity by the Se. These data, coupled with the rapid achievement of equilibrium when the As is first started, suggest that the turnover time for As in this mouse model is relatively short, on the order of 1 or 2 days.

No significant inhibitory effect of p-XSC was observed in UVR carcinogenesis, although vitamin E (presumably working as an antioxidant) produced a 20% decrease in UVR carcinogenesis (*p* ≤ 0.05). The lack of an Se effect on UVR carcinogenesis is consistent with the finding that the Se is acting very differently than vitamin E (i.e., blocking As rather than reducing oxidative damage directly). It also follows from the present results that little, if any, As was sequestered by the endogenous Se, presumably because most of the latter is normally locked inside of antioxidative and other enzymes (Das et al. 2004).

Much work has been published indicating that organic Se can act as an antioxidant and can interfere with carcinogens other than As; this has been shown particularly in the initiation phase of carcinogenesis by hydrocarbons (Das et al. 2004). Results with organoselenium (p-XSC) and vitamin E indicate that both organic Se and vitamin E protect against As(III)-enhanced UVR carcinogenesis in mouse skin and against 8-oxo-7,8-dihydro-2′-deoxyguanosine levels in epidermal DNA of the same mice (Uddin et al. 2005). However, there is no reason that anti-oxidative and sequestering mechanisms could not be simultaneously operational.

Overall, the results support the idea that Se inhibits the cocarcinogenic activity of As in skin by preventing the peripheral distribution of As from drinking water. In the mouse skin As is not carcinogenic by itself, but even if it were, Se would likely be a potent preventive agent. Evidence that Se prevents As from getting to peripheral tissues suggests that Se would be anticarcinogenic even in tissues where As is carcinogenic by itself.

The highly potent cocarcinogenic activity of As with UVR in mouse skin As (roughly a 5- or 6-fold enhancement) raises a serious question whether human studies showing that As is a carcinogen by itself may have overlooked a low-level companion (Burns et al. 2004; Rossman et al. 2004). Precedence for As as a cocarcinogen in humans has been derived from studies showing synergistic action with tobacco use for lung cancer induction in occupationally exposed workers (Choiu et al. 1995; Hertz-Picciotto et al. 1992) and with radon in tin miners (Xuan et al. 1993).

Although the mechanism of formation of As–Se complexes is not fully understood, current evidence indicates that exogenous As depletes glutathione pools and elevates arsenation of protein sulfhydryl groups (Haugen et al. 2004). The levels of several selenoenzymes, particularly glutathione peroxidase, are lowered by As, and Se supplementation affects the same enzymes oppositely (Miyazaki et al. 2005). Arsenic binds initially to the sulfur of glutathione in selenoenzymes, after which a rearrangement can produce a stable complex containing two molecules of glutathione and one atom each of As and Se (Gailer et al. 2002b; Manley et al. 2006).

The present study points to the use of dietary Se as a potentially effective countermeasure against any and all types of exposures to As in drinking water that may result in unhealthy outcomes, because the As is effectively sequestered and excreted in the presence of sufficient levels of Se.
